# Relationship Between Clinical Features and Body Mass Index Among Hypertensive Patients: A Cross-Sectional Study

**DOI:** 10.7759/cureus.11615

**Published:** 2020-11-22

**Authors:** Muhammad Yasir Paracha, Faran Khalid, Muhammad Adeel Qamar, Syed Liaquat Ali, Simran Singh, Umme Rubab, Adnan Anwar, Atif A Hashmi

**Affiliations:** 1 Cardiology, National Institute of Cardiovascular Diseases, Karachi, PAK; 2 Internal Medicine, Dow University Hospital, Dow University of Health Sciences, Karachi, PAK; 3 Biochemistry, Al-Tibri Medical College, Isra University, Karachi, PAK; 4 Internal Medicine, Jinnah Sindh Medical University, Karachi, PAK; 5 Physiotherapy, Jinnah Sindh Medical University, Karachi, PAK; 6 Physiology, Al-Tibri Medical College, Isra University, Karachi, PAK; 7 Pathology, Liaquat National Hospital and Medical College, Karachi, PAK

**Keywords:** body mass index, hypertension, clinical features, overweight, obesity

## Abstract

Objectives

Hypertension is strongly related to body mass index (BMI). Obesity has been the single main contributor to hypertension. Furthermore, the clinical manifestations are normally associated with BMI in hypertensive patients. This study aimed to evaluate the relationship between clinical features and BMI among hypertensive patients in both males and females.

Methodology

A retrospective cross-sectional study was conducted among 296 patients having a self-reported history of hypertension and on anti-hypertensive medication. The study was conducted in the medical outpatient department of a secondary care hospital in Karachi during six months (January 2020 to June 2020). A detailed history was taken from each patient about hypertension-related symptoms, and clinical examination was performed. Blood pressure was measured using a sphygmomanometer with a stethoscope.

Results

Of the 296 patients, 156 (52.2%) were males and 140 (47.3%) were females; 16 (5.4%) of them were underweight, 91 (30.7%) were normal weight, 129 (43.6%) were overweight, and 60 (20.3%) were obese; in addition, 106 (35.8%) reported edema and 71 (24.0%) reported nausea and so on. As far as the association of clinical features and BMI was concerned, our study results showed that only edema (p=0.017) and nausea (p=0.044) were significantly associated with the BMI of the patients. Patients with edema were more likely to be obese than those without edema (29.2% vs. 15.3%), whereas patients with nausea were more likely to be overweight than those without nausea (57.7% vs. 39.1%).

Conclusions

Our study showed that among the clinical features, edema and nausea were significantly associated with the BMI of the patients, whereas the relationship with others was insignificantly related to BMI of the patients in both male and female patients.

## Introduction

Hypertension (HTN), also known as raised or high blood pressure (HBP), is a condition in which the blood vessels have persistently elevated pressure [1]. If HBP remains for a longer period, it becomes a key risk factor for coronary artery diseases, heart failure, stroke, chronic renal diseases, dementia, and peripheral arterial disease [2]. HTN can be further divided into essential (primary) and secondary HTN. Around 90-95% of patients are primary, whereas other 5-10% are considered secondary HTN. Primary HTN is described as HBP because of irregular lifestyles and genetic factors [3]. The factors that increase the possibility of HTN include additional salt intake, overweight, smoking habits, junk foods, and the use of alcohol. Secondary HTN occurs through detectable cause, for instance, chronic kidney disease (CKD), narrow arteries of the kidneys, a disorder of the endocrine, or usage of birth control pills [4].

In addition, obesity and related cardiovascular, metabolic, and renal ailments have swiftly become a main risk to universal health [5]. Global obesity has approximately doubled since 1980 and up-to-date estimates show that more than 1.4 billion adults are overweight or obese. In the USA, more than 65% of adults are overweight and 36% are obese with a body mass index (BMI) of >30 kg/m^2^ [6]. Numerous countries have even greater obesity rates, for instance, the expected incidence of obesity among adults increased up to 50% for males in Tonga and for females in Libya, Kuwait, Kiribati, Samoa, and Qatar [7].

As far as studies in Pakistan are concerned, it has been reported that approximately 30% of HTN in patients occurs because of obesity in males up to 45 years; nevertheless, in few patients, it is as high as 60% [8]. One more study revealed in Pakistan that overweight and obesity, particularly in college and university students, is approximately 40% [9].

During various previous years, there are more elements that boost the pathophysiology of obesity accompanying HTN as metabolic illnesses and raised BPs are persistent and leading cause of dyslipidemia, target organ injury, and diabetes mellitus, leading to CKD [10]. Our objective, therefore,was to assess the relationship between clinical features with BMI among both the genders in hypertensive patients.

## Materials and methods

A retrospective cross-sectional study was conducted of patients having HTN, which was self-reported, and on anti-hypertensive medicines. A total of 296 patients aged 18 years or above were recruited in the study from the medical outpatient department of a secondary care hospital in Karachi using a consecutive sampling technique. The duration of the study was from January 2020 to June 2020.

A thorough history was recorded from every patient about HTN-related clinical features, and clinical examination was performed. Patients with a history of diabetes, cardiac events, neurological disorders, cluster headache, gastrointestinal disease, and morbid obesity were excluded from the study. Blood pressure was measured using a sphygmomanometer through stethoscope.

Data analysis was performed using Statistical Package for Social Sciences (SPSS) Version 26.0 (IBM Corp., Armonk, NY, USA). The descriptive analysis was expressed as frequency and percentages. After checking for normality, inferential analysis was executed using Spearman’s correlation. A p-value of <0.05 was considered as statistically significant.

## Results

Of the 296 patients, 156 (52.7%) were males and 140 (47.3%) were females; 16 (5.4%), 91 (30.7%), and 129 (43.6%), 60 (20.3%) of them were underweight, normal weight, overweight, and obese, respectively. Also, 196 (66.2%) of patients gave a positive history of headache, 146 (49.3%) reported vertigo, 106 (35.8%) reported edema, 120 (40.5%) reported chest pain, 140 (47.3%) reported vision problems, 139 (47.0%) reported dyspnea, 9 (3.0%) reported epistaxis, 112 (37.8%) reported increased urinary frequency, 71 (24.0%) reported nausea, 99 (33.4%) reported sleep apnea, 94 (31.8%) reported palpitation, 190 (64.2%) reported fatigue, and 161 (55.4%) reported confusion (Table 1).

**Table 1:** Patient Characteristics BMI, body mass index

Variables (n = 296)		Number (%)
Gender	Male	156 (52.7)
	Female	140 (47.3)
BMI categories	Underweight	16 (5.4)
	Normal weight	91 (30.7)
	Overweight	129 (43.6)
	Obese	60 (20.3)
Headache history	Yes	196 (66.2)
	No	100 (33.8)
Vertigo (dizziness)	Yes	146 (49.3)
	No	150 (50.7)
Edema (body swelling)	Yes	106 (35.8)
	No	190 (64.2)
Chest pain	Yes	120 (40.5)
	No	176 (59.5)
Vision problems	Yes	140 (47.3)
	No	156 (52.7)
Dyspnea (difficulty in breathing)	Yes	139 (47.0)
	No	157 (53.0)
Epistaxis	Yes	9 (3.0)
	No	287 (97.0)
Increased urinary frequency	Yes	112 (37.8)
	No	184 (62.2)
Nausea (feeling of vomit)	Yes	71 (24.0)
	No	225 (76.0)
Sleep apnea (inability to sleep)	Yes	99 (33.4)
	No	197 (66.6)
Palpitation	Yes	94 (31.8)
	No	202 (68.2)
Fatigue (weakness or tiredness)	Yes	190 (64.2)
	No	106 (35.8)
Confusion	Yes	161 (54.4)
	No	135 (45.6)

Further analysis revealed that among clinical features, only edema (p=0.017) and nausea (p=0.044) were substantially linked to BMI of patients; patients with edema were more likely to be obese than those without edema (29.2% vs. 15.3%), whereas patients with nausea were more likely to overweight than those without nausea (57.7% vs. 39.1%). None of the other clinical features were significantly associated with the BMI of the patients (Table 2).

**Table 2:** Association of Clinical Features with BMI BMI, body mass index

Variables (n = 296)		BMI Categories				p-Value
		Underweight	Normal Weight	Overweight	Obese	
		Count (%)	Count (%)	Count (%)	Count (%)	
History of headache	Yes	8 (4.1)	62 (31.6)	86 (43.9)	40 (20.4)	0.563
	No	8 (8.0)	29 (29.0)	43 (43.0)	20 (20.0)	
Vertigo	Yes	9 (6.2)	43 (29.5)	61 (41.8)	33 (22.6)	0.694
	No	7 (4.7)	48 (32.0)	68 (45.3)	27 (18.0)	
Edema	Yes	4 (3.8)	25 (23.6)	46 (43.4)	31 (29.2)	0.017
	No	12 (6.3)	66 (34.7)	83 (43.7)	29 (15.3)	
Chest pain	Yes	7 (5.8)	34 (28.3)	55 (45.8)	24 (20.0)	0.875
	No	9 (5.1)	57 (32.4)	74 (42.0)	36 (20.5)	
Vision problems	Yes	8 (5.7)	48 (34.3)	58 (41.4)	26 (18.6)	0.617
	No	8 (5.1)	43 (27.6)	71 (45.5)	34 (21.8)	
Dyspnea	Yes	8 (5.8)	43 (30.9)	63 (45.3)	25 (18.0)	0.821
	No	8 (5.1)	48 (30.6)	66 (42.0)	35 (22.3)	
Epistaxis	Yes	Nil	3 (33.3)	2 (22.2)	4 (44.4)	0.244
	No	16 (5.6)	88 (30.7)	127 (44.3)	56 (19.5)	
Increased urinary frequency	Yes	8 (7.1)	35 (31.2)	53 (47.3)	16 (14.3)	0.188
	No	8 (4.3)	56 (30.4)	76 (41.3)	44 (23.9)	
Nausea	Yes	2 (2.8)	18 (25.4)	41 (57.7)	10 (14.1)	0.044
	No	14 (6.2)	73 (32.4)	88 (39.1)	50 (22.2)	
Sleep apnea	Yes	4 (4.0)	33 (33.3)	38 (38.4)	24 (24.2)	0.405
	No	12 (6.1)	58 (29.4)	91 (46.2)	36 (18.3)	
Palpitation	Yes	5 (5.3)	31 (33.3)	41 (43.6)	17 (18.1)	0.908
	No	11 (5.4)	60 (29.7)	88 (43.6)	43 (21.3)	
Fatigue	Yes	8 (4.2)	60 (31.6)	79 (41.6)	43 (22.6)	0.325
	No	8 (7.5)	31 (29.2)	50 (47.2)	17 (16.0)	
Confusion	Yes	10 (6.2)	46 (28.6)	68 (42.2)	37 (23.0)	0.495
	No	6 (4.4)	45 (33.3)	61 (45.2)	23 (17.0)	

## Discussion

Our study demonstrated that only edema and nausea were significantly associated with BMI among hypertensive patients. Literature review revealed a significant correlation of HTN with BMI even after regulating for other covariates and suggested that overweight and obesity are the main causes of progress of HTN and play a vital role in its pathogenesis [11]. According to a study conducted on adults in Europe, the propensity to be overweight was between 32% and 79% in men and 28% and 78% in women, whereas obesity incidence was between 5% and 23% in men and 7% and 36% in women [12]. In our study, obesity prevalence was 156 (52.7%), which proved to be 0.6-fold higher in men than in females (140; 47.3%) and which is consistent with the literature. Higher obesity level in women than in men is due to physiological and biological causes, for example, sedentary living style, fat diet, high-calorie food intake, pregnancy, parity, breast-feeding duration, marriage, and menopause [13].

A study by Gupta and Kapoor [14] revealed that the prevalence of HTN was higher in males than in females for all the categories of BMI. Even though females were more obese than males, males were at high risk for HTN in that study. The results of our study were consistent with the aforementioned study and showed that 129 (43.6%) males with high BMI category had HTN compared to females.

Headache, epistaxis, faintness, psychomotor agitation, chest pain, dyspnea, neurologic deficits, and paresthesias are the clinical features exhibited in HTN. Sometimes, the patient might complain of epigastric pain and vomiting; therefore, upon investigation, abrupt reflexes of the tendons, and edema of hands, face, or feet can be identified [15]. These findings were also consistent with our study in which 86 (43.9%) cases with high BMI had a history of headache, with p= 0.563 that was statistically insignificant. While 61 (41.8%) cases with a high BMI had vertigo, with p= 0.694 that was statistically insignificant, chest pain, dyspnea, and increased urinary frequency were mostly found in those with high BMI. On the other hand, edema was present in 46 (43.4%) cases with high BMI, with p=0.017 that was statistically significant, and nausea was found in 41 (57.7%) cases with high BMI, with p=0.044 that was statistically significant.

Hypertensive encephalopathy (headache, convulsion, and confusion) is due to elevated blood pressure that affects the blood-brain barrier that weakens its functioning, resulting in cerebral hyperperfusion and ultimately leading to brain edema [15]. Our study showed that 68 (42.2%) cases with high BMI were in a state of confusion, with p=0.495 that was statistically insignificant.

Tassaduqe et al. found an elevated occurrence of HTN with increasing age [16]. However, Humayun et al. reported that BMI in hypertensive patients has a strong association with age [17].

Our study was also consistent with past studies that revealed that HTN was correlated with BMI rather than age. Tesfaye et al. conducted research among Asians and Africans and reported that systolic blood pressure (SBP) and diastolic blood pressure (DBP) were positively correlated with age, whereas BMI was negatively correlated with age in some patients [18]. In contrast, our study findings are dissimilar to the study by Huang et al., in which females with high BMI had a significant pervasiveness of HTN compared with males in the same group [19].

The results of our study, in accordance with other same studies, provide beneficial clinical statistics when measuring, handling, and counselling patients with HTN. Furthermore, these interpretations and more studies are needed taking into account more aspects and features to determine the impact of weight reduction on the occurrence or severity of HTN-related manifestations.

## Conclusions

Our study showed that among the clinical features, only edema and nausea were significantly associated with the BMI of the patients. Patients with edema were more likely to be obese than those without edema, whereas patients with nausea were more expected to have overweight than those without nausea. However, the relationships with other features were insignificantly related to the BMI of the patients in both male and female patients.

## References

[1] Naish J, Court DS (2014). Medical Sciences. Medical sciences.

[2] Lackland DT, Weber MA (2015). Global burden of cardiovascular disease and stroke: hypertension at the core. Can J Cardiol.

[3] Poulter NR, Prabhakaran D, Caulfield M (2015). Hypertension. Lancet.

[4] James PA, Oparil S, Carter BL (2014). 2014 evidence-based guideline for the management of high blood pressure in adults: report from the panel members appointed to the Eighth Joint National Committee (JNC 8). JAMA.

[5] Hall JE, da Silva AA, do Carmo JM (2010). Obesity-induced hypertension: role of sympathetic nervous system, leptin, and melanocortins. J Biol Chem.

[6] Ogden CL, Carroll MD, Kit BK, Flegal KM (2014). Prevalence of childhood and adult obesity in the United States, 2011-2012. JAMA.

[7] Murray CJ, Ortblad KF, Guinovart C (2014). Global, regional, and national incidence and mortality for HIV, tuberculosis, and malaria during 1990-2013: a systematic analysis for the Global Burden of Disease Study 2013. Lancet.

[8] Jafar TH, Chaturvedi N, Pappas G (2006). Prevalence of overweight and obesity and their association with hypertension and diabetes mellitus in an Indo-Asian population. CMAJ.

[9] Shams N, Niaz F, Motwani R, Shaikh Z, Saleem F (2015). Obesity and hypertension in female medical students; frequency and risk factors. J Liaquat Uni Med Health Sci.

[10] Hall ME, do Carmo JM, da Silva AA, Juncos LA, Wang Z, Hall JE (2014). Obesity, hypertension, and chronic kidney disease. Int J Nephrol Renovasc Dis.

[11] Sullivan CA, Kahn SE, Fujimoto WY, Hayashi T, Leonetti DL, Boyko EJ. (2015). Change in intra-abdominal fat predicts the risk of hypertension in Japanese Americans. Hypertension.

[12] (2020). Obesity and Overweight. https://www.who.int/news-room/fact-sheets/detail/obesity-and-overweight.

[13] Gavin AR, Simon GE, Ludman EJ (2010). The association between obesity, depression, and educational attainment in women: the mediating role of body image dissatisfaction. J Psychosom Res.

[14] Gupta S, Kapoor S (2010 Autumn). Sex differences in blood pressure levels and its association with obesity indices: who is at greater risk. Ethn Dis.

[15] Alshami A, Romero C, Avila A, Varon J (2018). Management of hypertensive crises in the elderly. J Geriatr Cardiol.

[16] Tassaduqe K, Ali M, Salam A (2004). Hypertension in relation to obesity, smoking, stress, family history, age and marital status among human population of Multan, Pakistan. J Med Sci.

[17] Humayun A, Shah AS, Sultana R (2009). Relation of hypertension with body mass index and age in male and female population of Peshawar, Pakistan. J Ayub Med Coll Abbottabad.

[18] Tesfaye F, Nawi NG, Van Minh H, Byass P, Berhane Y, Bonita R, Wall S (2007). Association between body mass index and blood pressure across three populations in Africa and Asia. J Hum Hypertens.

[19] Huang Z, Willett WC, Manson JE, Rosner B, Stampfer MJ, Speizer FE, Colditz GA (1998). Body weight, weight change, and risk for hypertension in women. Ann Intern Med.

